# Application of Nanotechnology in Analysis and Removal of Heavy Metals in Food and Water Resources

**DOI:** 10.3390/nano11071792

**Published:** 2021-07-09

**Authors:** Zhaoyuan Gong, Hiu Ting Chan, Qilei Chen, Hubiao Chen

**Affiliations:** 1 School of Chinese Medicine, Hong Kong Baptist University, Hong Kong 999077, China; 19428898@life.hkbu.edu.hk (Z.G.); kristychan@hkbu.edu.hk (H.T.C.); 2 Institute of Basic Research in Clinical Medicine, China Academy of Chinese Medical Sciences, Beijing 100700, China

**Keywords:** nanotechnology, heavy metal, food safety, water safety, nanomaterials

## Abstract

Toxic heavy metal contamination in food and water from environmental pollution is a significant public health issue. Heavy metals do not biodegrade easily yet can be enriched hundreds of times by biological magnification, where toxic substances move up the food chain and eventually enter the human body. Nanotechnology as an emerging field has provided significant improvement in heavy metal analysis and removal from complex matrices. Various techniques have been adapted based on nanomaterials for heavy metal analysis, such as electrochemical, colorimetric, fluorescent, and biosensing technology. Multiple categories of nanomaterials have been utilized for heavy metal removal, such as metal oxide nanoparticles, magnetic nanoparticles, graphene and derivatives, and carbon nanotubes. Nanotechnology-based heavy metal analysis and removal from food and water resources has the advantages of wide linear range, low detection and quantification limits, high sensitivity, and good selectivity. There is a need for easy and safe field application of nanomaterial-based approaches.

## 1. Introduction

Heavy metals generally have densities of more than 5 g/cm^3^. Most heavy metals, such as arsenic, lead, mercury, chromium, zinc, cadmium, copper, and nickel, are regarded as environmental pollutants [1]. Since heavy metals are not biodegradable, heavy metal ions in waters and in soil may be biologically accumulated via the food chain towards the human body [2]. Other than the essential elements needed for human body, such as sodium, calcium, and zinc, some heavy metals exert serious toxicities even at low intake levels (Table 1). However, heavy metals such as copper, ion, cobalt, and chromium in small concentrations are essential elements in the human body. Therefore, it is necessary to detect and remove heavy metals from our food and water resources.

Conventional approaches for heavy metal detection from complex matrices include atomic absorption spectroscopy [3], neutron activation analysis [4], X-ray fluorescence spectrometry [5], energy dispersive X-ray fluorescence [6], inductively coupled plasma mass spectroscopy [7,8], inductively coupled plasma atomic/optical emission spectrometry [9], and flame atomic absorption spectrometry [10]. Although they can generally achieve satisfactory detection sensitivity, they are inevitably challenged with heavy equipment, high cost, and lab-only and complicated operation. Classical methods for heavy metal removal from wastewater include precipitation, ion exchange [11], reverse osmosis [12], membrane filtration [13] and oxidation [14]. However, their effectiveness is limited due to disadvantages of sludge contamination, high pH sensitivity, and corrosiveness [15,16,17].

Nanotechnology refers to employing multidisciplinary techniques integrating principles of physics, chemistry, engineering, and biology to build nanoscale materials or devices [18]. With the upsurge of nanotechnology, nanomaterials have been delicately designed and fabricated for heavy metal detection and removal, exerting numerous advantages as compared to the previous methods.

**Table 1:** Representative toxic heavy metals and their adverse effects on human health.

Element	Adverse Effects on Human Health	Ref.
Lead (Pb)	Kidney damage, cognitive disorders, hypertension.	[19]
Mercury (Hg)	Maternal and fetal toxicity; mental retardation, cerebral palsy, deafness, blindness, dysarthria; gastrointestinal tract toxicity: necrotic intestinal mucosa, abdominal pain, vomiting, bloody diarrhea.	[20,21]
Arsenic (As)	Skin lesions, blackfoot disease, peripheral neuropathy, encephalopathy, hepatomegaly, liver cirrhosis, altered heme metabolism, myeloid depression, diabetes, proximal tubule degeneration, papillary and cortical necrosis.	[22,23]
Chromium (Cr)	Skin rashes, stomach ulcers, weakened immune systems, kidney and liver damage, genetic material change, lung cancer; respiratory system issues: rhinitis, pharyngitis, laryngitis, bronchitis.	[24]
Copper (Cu)	Wilsons’s disease, upper respiratory tract irritation, nasal mucosa, hemolytic anemia, epigastric pain, nausea, dizziness.	[25]
Nickel (Ni)	Pneumoconiosis, asthma, lung and laryngeal cancer.	[26]
Zinc (Zn)	Tachycardia, vascular shock, dyspepsia, nausea, headache, nasal and lung cancer, asthma, vomiting, diarrhea, hypoglycemia, pancreatitis and liver parenchyma damage, growth and reproduction disorders.	[27]
Cadmium (Cd)	Kidney (proximal renal tubule cells), lung, liver, and vascular system damage; bone demineralization.	[28]

## 2. Nanotechnology-Based Heavy Metal Analysis in Food and Water

### 2.1. Electrochemical Analysis by Nanosensors

#### 2.1.1. Carbon-Based Nanomaterials

**Figure 1:**
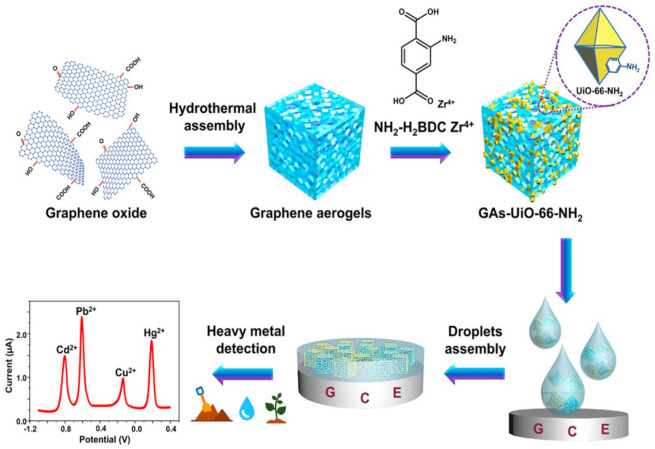
Schematic Illustration of GAs-UiO-66-NH_2_ Composite Synthesized via the In Situ Growth of the UiO-66-NH_2_ Crystal on GAs Matrix and the Detection of Heavy-Metal Ions by GAs-UiO-66-NH_2_ Modified Electrode [34]. Reproduced with permission from Lu et al., *Anal. Chem.*; published by the American Chemical Society, 2018.

Graphene, graphene oxides (GO), and reduced graphene oxide (rGO), all with heavy metal ion adsorption capability, have been developed as advanced nano-electrocatalyst for constructing electrochemical sensors (Table 2). Graphene-based electrodes coated with in-situ bismuth film [29] and electrostatically attracted phytic acid [30], respectively, were reported with boosted sensitivity towards Pb^2+^ and Cd^2+^. Polypyrrole (PPy), a conductive polymer, can be physically adsorbed on GO with good dispersion [31] and subsequently enhance electrode conductivity [32]. Nanocomposite of GO linked with a bipyridine ligand, [Ru(bpy)_3_]^2+^, was used to modify a gold (Au) electrode with enhanced mass transport efficiency and charge transfer, which favored the sensitive and selective detection of metal ions [33]. Moreover, as a novel porous derivative of GO, graphene aerogel (GA), has unique porous structure which provides abundant coupling locations with metal–organic frameworks (MOFs). A GA-MOF composite-modified glass carbon electrode (GCE) showed enhanced conductivity and high affinity to heavy metals ions via hydrophilic groups from MOF; the GA-MOF-based GCE was demonstrated with simultaneous detection of various heavy metal ions in river water, soil, and spinach (Figure 1) [34].

Various rGO-based electrodes have been applied in food and water samples. rGO has strong hydrophilicity and strong adsorption capacity for metal ions because of the functional groups on its surface [35]. An Au electrode covered by micro-patterned rGO and electrodeposited bismuth was applied in trace analysis of Cd^2+^ and Pb^2+^ in drinking water [36]. Green hybrid electrochemical nanosensors constructed with rGO, cellulose nano whiskers (promoted green reduction of GO resulting in rGO), and polyamide 6 electrospun nanofibers (increased electrode surface area and amplified signal) were applied in detecting heavy metals in water samples [37,38].

Carbon nanotubes (CNTs), cylindrical macromolecules with carbon atoms arranged in hexagonal rings, have significant advantages of ordered structure, high ground object ratio, good mechanical strength, and high electrical conductivity [39]. Single-walled CNTs (SWCNTs) have high sensitivity to local dielectric or redox environment due to their band gap; they also specially undergo strong redox reaction with Hg^2+^. SWCNT-based nanosensors with good sensitivity and selectivity were successfully prepared, for instance, to analyze drinking water samples [40]. Multi-walled CNTs (MWCNTs) have become one of the most active materials in carbon paste electrodes due to their unique structure. A super selectivity sensor was reported comprising MWCNTs (as a carbon paste electrode), 1-*n*-butyl-3-methylimidazolium tetrafluoroborate (BMIM·BF_4_; as a super conductive binder), and 1-(2-ethoxyphenyl)-3-(3-nitrophenyl) triazene (ENTZ; as an ionophore); the sensor showed greatly enhanced potentiometric sensitivity, good reproducibility, low response time and long-term stability in quantification of Hg^2+^ in water samples [41].

When designing carbon-based sensing electrodes for detecting heavy metal ions, the combination of CNTs and graphene derivatives works better than either alone. A study used graphene-MWCNTs to prepare nanocomposites with excellent conductivity were obtained conveniently by the direct electrochemical reduction of GO–MWCNTs nanocomposites. The graphene-MWCNTs-modified electrode was successfully applied to the simultaneous detection of real electroplating effluent samples containing a large number of surface-active impurities [42]. In another study, 2-aminothiophene (L)-grafted carboxylate-functionalized MWCNTs (L-g-MWCNT-COOH) were synthesized as a sensor with hydrophobic environment and high complex stability, due to the presence of hard donor atoms (N, O, and S) and aromatic rings. The sensor was applied to various environmental samples [43].

Ordered mesoporous carbon nitride (MCN) as the platform of electrochemical sensor has great detection sensitivity of phenols and heavy metals [44]; self-doped polyanilines (SPANs) have strong conductivity and electrochemical activity. Therefore, SPAN/MCN-modified GCE, combining advantages of SPAN and MCN, has been reported as a novel electrode for heavy metal analysis in river water samples [45]. Graphitized carbon nitride (g-C_3_N_4_), a 2D structure composed of tri-s-triazine units connected with planar amino groups, with high affinity to metal ions due to its electrostatic bonding or coordination with several N atoms [46], is widely applied as green material for its simple preparation, good chemical stability and high catalytic activity [47]. An electrode modified by poly (2,5-bis(3,4-ethylenedioxythienyl) pyridine) (poly (BPE)) and g-C_3_N_4_ demonstrated to enhance adsorption of metal ions from tap water; the binding of poly(BPE) to g-C_3_N_4_ not only improved the surface conduction pathway of the electrode, but also produced a strong conjugation effect [48]. Colloidal hollow carbon nanospheres (HCNs) as sensing materials have distinctive features of large void and porous shell configuration, special spherical morphology, high specific surface area, and low density. A series of carbon electrodes with good microstructure were prepared by calculating contact points between HCNs and GCE, to bridge the electrode structure with sensing performance; such sensors were proved successful in analyzing heavy metals in drinking water [49].

#### 2.1.2. Magnetic Nanoparticles

Magnetic nanoparticles (MNPs), such as superparamagnetic metal oxides, are commonly used as electrode modifiers, because they can significantly enhance electron strength transfer between the electrode and the analyte, leading to a high charge transfer capability [50]. Chitosan (CHT) is a natural polymer with abundant primary amino and hydroxyl groups; it has excellent adhesion, hydrophilicity, doping feasibility, and chemical stability. A novel electrode was coated with a mixture of Fe_3_O_4_ and CHT solution on an ionic liquid solid phase extraction, followed by a bismuth film deposited in situ; such electrode combined the advantages of Fe_3_O_4_, CHT, and ionic liquid screen-printed electrode (ILSPE), and accurately detected the content of Cd^2+^ in soil samples thus reducing the contamination of cultivated rice [51].

#### 2.1.3. Semiconducting Nanomaterials

Tin oxide (SnO_2_) has well-known semi conductivity; however, SnO_2_ nanoparticles tend to aggregate easily, hindering their optimal performance. rGO can serve as a template to prevent the accumulation of SnO_2_ nanoparticles. rGO/SnO_2_ electrodes were reported with applications of synchronous and selective electrochemical detection of trace Cd^2+^, Pb^2+^, Cu^2+^, and Hg^2+^ in drinking water [52].

**Figure 2:**
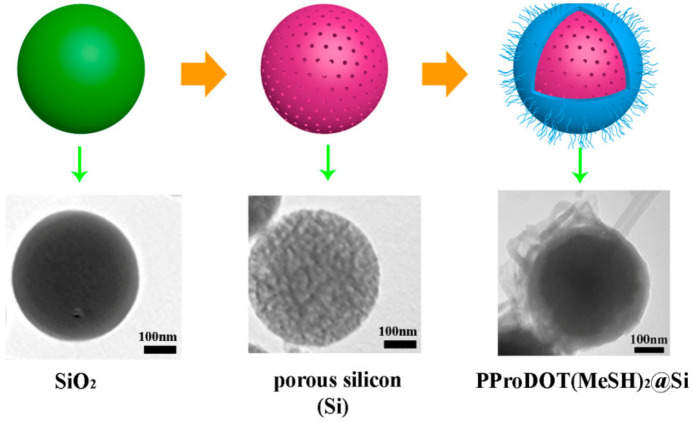
The synthesis route of PProDOT(MeSH)_2_@Si composite [56]. Reproduced with permission from Abdulla et al., *Polymers (Basel)*; published by the MDPI, 2019.

Porous silicon has been reported to be a nano scavenger for determining trace level of heavy metals in water. Thiol- and amino-grafted Si nanowire-modified electrodes exhibit poor electrochemical stability and sensitivity [53], such defects can be overcome by combining Si with a conductive polymer, e.g. poly (3,4-ethyldioxythiophene) (PEDOT). PEDOT and its derivatives are typical electrode sensor materials, with low oxidation potential and high thermal and chemical stability. The polymers also contained a variety of special functional groups and donor atoms that enhanced their coordination ability with metal ions [54]. Similarly, poly(3,4-proplenedioxythiophene) (PProDOT) and its derivatives showed capability in detecting heavy metal ions [55] that thiol-grafted (3,4-proplenedioxythiophene) (PProDOT(MeSH)_2_)/Si composites significantly enhanced heavy metal adsorption and improved the performance of catalytic electrode materials in analyzing water samples (Figure 2) [56].

#### 2.1.4. Noble Metal Nanoparticles

Gold nanoparticles (AuNPs) have been demonstrated to be appropriate to modify electrodes due to their small size, good electrical conductivity and excellent catalytic activity, based on which they can “guide” electroactive substances towards the electrodes. AuNP-modified GCE with chloride desorption can provide a three-times higher sensitivity in trace detection of Hg^2+^ than that with no desorption step. Such detection was proven successful in analyzing groundwater samples; the results were compared well with those obtained by conventional cold vapor atomic fluorescence spectroscopy [57].

**Table 2:** Representative studies of electrochemical analysis of heavy metals from food and water resources by nanosensors.

Electrode	Analytical Technique	Sample	Linear Range	Limit of Detection	Ref.
Graphene/Bi/PGE	SWASV	Canadian-certified reference water sample and tap water sample	5–100 μg/L Cd^2+^; Pb^2+^	0.12 μg/L Cd^2+^ 0.29 μg/L Pb^2+^	[29]
PA/PPy/GO	DPV	Tap water samples	5–150 μg/L Cd^2+^; Pb^2+^	2.13 μg/L Cd^2+^ 0.41 μg/L Pb^2+^	[30]
[Ru(bpy)_3_]^2+^/GO/AuNPs	DPV	Cauvery river water	0.02–3 μM Cd^2+^; Pb^2+^, 0.02–2 μM As^3+^; Hg^2+^	2.8 nM Cd^2+^ 1.41 nM Pb^2+^ 2.3 nM As^3+^ 1.6 nM Hg^2+^	[33]
MOF/GA/GCE	DPV	River water soil and vegetable sample (spinach)	0.01−1.5 μM Cd^2+^, 0.001−2 μM Pb^2+^, 0.01−1.6 μM Cu^2+^, 0.001−2.2 μM Hg^2+^	0.02 μM Cd^2+^ 1.5 nM Pb^2+^ 7 nM Cu^2+^ 2 nM Hg^2+^	[34]
Bi/rGO/Au electrode	SWASV	Drinking water sample	1.0–120.0 μg/L Cd^2+^; Pb^2+^	0.4 μg/L Pb^2+^ 1.0 μg/L Cd^2+^	[36]
PA6/CNW/rGO	DPV	Water sample	2.5–200 μM Hg^2+^	5.2 nM Hg^2+^	[38]
SWCNTs/SiO_2_	ISE	Drinking water sample	10 nM−1 mM Hg^2+^	10 nM Hg^2+^	[40]
BMIM·BF_4_/MWCNTs/graphite powder	ISE	Water sample	5.0 nM−1.0mM Hg^2+^	2.5 nM Hg^2+^	[41]
Graphene/MWCNTs/Bi	SWASV	Real electroplating effluent samples	0.5–30 mg/L Pb^2+^; Cd^2+^	0.2 μg/L Pb^2+^ 0.1 μg/L Cd^2+^	[42]
L-g-MWCNTs/CPE	ISE	Soils, waste waters, lead accumulator waste and black tea	5.9 nM − 1.0 mM Pb^2+^	3.2 nM Pb^2+^	[43]
SPANs/MCN/GCE	SWASV	River water sample	5–80 nM Cd^2+^; Pb^2+^	0.7 nM Cd^2+^ 0.2 nM Pb^2+^	[45]
Poly (BPE)/g-C_3_N_4_	DPV	Tap water samples	0.12–7.2 μM Cd^2+^, 0.08–7.2 μM Pb^2+^,	0.018 μM Cd^2+^ 0.00324 μM Pb^2+^	[48]
HCNs/GCE	SWASV	Drinking water sample	/	0.6 nM Pb^2+^	[49]
Fe_3_O_4_/Bi/ILSPE	DPV	Soil sample	0.5−40 μg/L Cd^2+^	0.05 μg/L Cd^2+^	[51]
SnO_2_/rGO/GCE	SWASV	Drinking water sample	0.3–1.2 μM Hg^2+^; Cu^2+^; Cd^2+^; Pb^2+^	0.3 nM Hg^2+^ 0.2 nM Cu^2+^ 0.1 nM Cd^2+^ 0.2 nM Pb^2+^	[52]
PProDOT (MeSH)_2_/Si/GCE	DPV	Tap water sample	0.04−2.8 μM Cd^2+^, 0.024−2.8 μM Pb^2+^, 0.16−3.2 μM Hg^2+^	0.00575 μM Cd^2+^ 0.0027 μM Pb^2+^ 0.0017 μM Hg^2+^	[56]
AuNPs/GCE	SWASV	Natural groundwater sample	0.3–10 nM Hg^2+^	80 pM Hg^2+^	[57]

Abbreviations: Square wave anodic stripping voltammetry (SWASV), differential pulse voltammetry (DPV), and ion-selective electrode (ISE), pencil graphite electrode (PGE), phytic acid (PA), polypyrrole (PPy), graphene oxide (GO), Au nanoparticles (AuNPs), metal–organic frameworks (MOFs), graphene aerogel (GA), glassy carbon electrode (GCE), reduced graphene oxide (rGO), polyamide 6 (PA6), cellulose nano whiskers (CNW), single-walled CNTs (SWCNTs), 1-*n*-butyl-3-methylimidazolium tetrafluoroborate (BMIM·BF4), multi-walled CNTs (MWCNTs), carbon paste electrode (CPE), self-doped polyanilines (SPANs), mesoporous carbon nitride (MCN), poly (2,5-bis(3,4-ethylenedioxythienyl)pyridine) (poly (BPE)), hollow carbon nanospheres (HCNs), ionic liquid screen-printed electrode (ILSPE), tin oxide (SnO_2_), thiol (–SH) grafted poly(3,4-proplenedioxythiophene) (PProDOT(MeSH)_2_), silicon spheres (Si).

### 2.2. Colorimetric Analysis Based on Gold and Silver Nanoparticles

#### 2.2.1. Gold Nanoparticles

**Figure 3:**
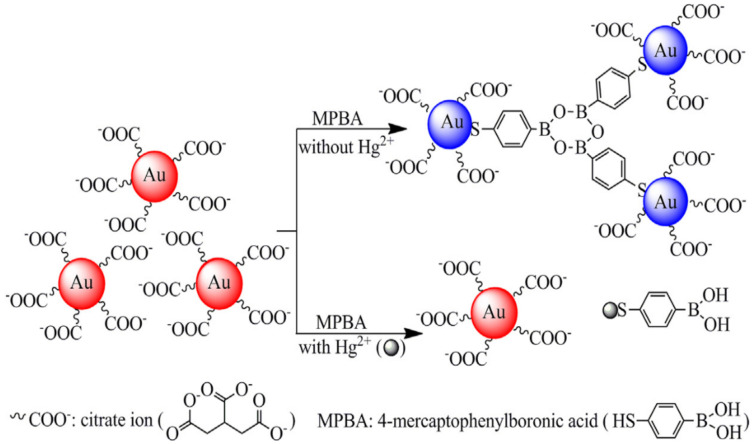
Schematic presentation of the MPBA/AuNPs colorimetric mechanism for Hg^2+^ detection [61]. Reproduced with permission from Zhou et al., *Sensors Actuators, B Chem.* published by the Elsevier B.V., 2014.

Since AuNPs have localized surface plasmon resonances (LSPR), some heavy metal ions can be analyzed based on dispersion of AuNPs [58] (Table 3). An Hg^2+^ sensor was reported constructed with 4-mercaptophenylboronic acid (MPBA) and AuNPs. The AuNPs can be initially stabilized with a coating of negatively charged citrate ions, which provide electrostatic repulsion preventing particle aggregation [59]. MPBA, an aggregation agent, exhibits a high affinity for AuNPs via Au–S crosslinking, leading to a visible color change of the AuNP solution from red to blue. With increasing concentration of Hg^2+^, the thiol group of MPBA would prefer binding with Hg^2+^ than polymerizing AuNPs, revealing the solution from blue to red [60]. Such Hg^2+^ nanosensors with high detection specificity were successfully applied in testing water samples [61] (Figure 3).

Besides particle dispersion, thiol-dependent aggregation of AuNPs can be utilized for detecting heavy metal ions, e.g. Co^2+^, Pb^2+^, Hg^+^, and Ag^+^. Co^2+^ level in aqueous solution can be measured based on aggregation of thiosulfate (S_2_O_3_^2−^)-stabilized AuNPs with the addition of ethylenediamine (en), a chelating agent that forms complexes with heavy metal ions. In the presence of S_2_O_3_^2−^ coating on AuNP surface, the Co^2+^/en complexes can be oxidized to Co(en)_3_^3+^ and in turn form (en)_2_CoS_2_O_3_^+^. The AuNPs will then aggregate due to insufficient surface charges, changing the solution from red to blue. Such label-free colorimetric sensing was adopted in rapid detection of Co^2+^ in drinking water and development of Co^2+^ test papers [62]. Similarly, in the case of measuring Pb^2+^ in environmental samples, the Pb/Au alloy formed on the surface of AuNPs would weaken the stability of Au^+^/S_2_O_3_^2−^ and thus enhance the access of 4-mercaptobutanol (4-MB) to AuNP surface, inducing AuNP aggregation [63]. When it comes to measuring aqueous Hg^2+^ and Ag^+^, AuNPs simultaneously coated with citric acid and Tween 20 can be used. The modified AuNPs are initially stable against high ionic strength; the citrate ions would induce bond formation of Hg–Au and Hg–Ag on AuNP surface, causing Tween 20 removal and consequently AuNP aggregation [64]. Furthermore, other thiol-containing substances, such as mercaptan carboxylic acid [65], quaternary ammonium compound, cysteine [66], dithioerythritol [67], and alkanethiols [68] have been reported in colorimetric detection for Hg^2+^.

Additionally, AuNPs can be capped by graphite-like nitride doped carbon quantum dots (AuNPs/g-CQDs) as a sensitive color-tuning indicator to measure the typical weak catalytic heavy metal ions of Cd^2+^ ions. Due to the large number of functional groups on the AuNPs/g-CQDs surface, the nanocomposite aggregates upon contacting Cd^2+^ ions. In addition, the biodistribution and aggregation of Cd^2+^ ions in mouse organ tissues have also been effectively detected, indicating that it has great potential in practical application [69].

Furthermore, leaching of AuNPs can be accelerated by GO in the presence of Pb^2+^, resulting in a color change. Such principle has been adopted in detecting Pb^2+^ in drinking water and river water samples [70].

#### 2.2.2. Silver Nanoparticles

Modified AgNPs have been reported as heavy metal sensors based on different detection principles, including color changes due to (1) particle aggregation, (2) increased fluorescence intensity, and (3) silver chloride precipitate formation.

The detection of Cu^2+^ can be performed based on induced aggregation of thiomalic acid (TMA)-functionalized AgNPs. In the presence of Cu^2+^, carboxylic acid groups in TMA preferentially interact with Cu^2+^ to form stable complexes, inducing aggregation of the AgNPs to generate a red shift in LSPR band. Such sensors have been applied in real-time monitoring of Cu^2+^ in various environmental water samples [71]. Similarly, 5-sulfosalicylic acid -functionalized AgNPs (SAA/AgNPs) is widely used as a low-cost ($0.10 per sample) sensor in detecting Cd^2+^ ions, for example, in tap water, milk, urine, and serum samples [72]. Xylan-capped AgNPs can easily react with Hg^2+^; formation of Hg^2+^/xylan complex results in nanoparticle aggregation. Therefore, xylan-AgNPs composites can detect Hg^2+^ with high sensitivity and selectivity, as a simple and fast approach for detection of Hg^2+^ in environmental water [73].

Poly(acrylic acid)-templated silver nanoclusters (PAA/AgNCs) can measure Hg^2+^ ions via selective fluorescence initiation. In the presence of Hg^2+^ ions, the fluorescence intensity of AgNCs would raise dramatically, causing the solution to change color from purple to light red, which can be observed by naked eye. The method has been proved practical in analyzing samples of river water, tap water, and mineral water [74].

An AgCl/Ag sensor modified by natural seaweed polysaccharide carrageenan was reported exhibiting sensitive and robust detection of Hg^2+^ ions from environmental or biological samples, where AgCl/Ag interacted with Hg^2+^ ions to form an Hg–Ag alloy which reduced surface plasmon resonance (SPR) intensity, and the formation of silver chloride caused a visible change in solution from dark brown to white [75]. AgNPs can also be synthesized using *Cordia myxa* leaf extract for determination of Hg^2+^, where Ag atoms can be selectively oxidized by Hg^2+^, leading to AgNPs dissolution in the presence of Cl^−^ ions [76].

**Table 3:** Representative studies of colorimetric analysis of heavy metals from food and water resources based on nanoparticle aggregation.

Electrode	Sample	Linear Range	Limits of Detection	Ref.
MPBA/AuNPs	Water samples	0.01–5 μM Hg^2+^	8 nM Hg^2+^	[61]
(en)/S_2_O_3_^2−^/AuNPs	Drinking water	0.1–0.7 μM Co^2+^	0.04 μM (2.36 ppb) Co^2+^	[62]
4-MB/AuNPs (S_2_O_3_^2−^)	River water, Montana soil, and urine samples	0.5–10 nM Pb^2+^	0.2 nM Pb^2+^	[63]
Tween 20/Citrate ions/AuNPs	Drinking water and seawater sample	/	0.1 μM Hg^2+^; Ag^+^	[64]
DTETAuNPs	Water sample	1–6 μM Hg^2+^	100 nM Hg^2+^	[67]
AuNPs/g-CQDs	Mice organ tissues sample	0.01–3.0 μM Cd^2+^	10 nM Cd^2+^	[69]
AuNPs/GO/S_2_O_3_^2−^	Pure drinking water and river water samples	0.1–20 μM Pb^2+^	0.05 μM Pb^2+^	[70]
TMA/AgNPs	Tap, lake, and river water sample	/	0.5 nM Cu^2+^	[71]
SAA/AgNPs	Tap water, lake water, milk, urine, and serum sample	0.05–1.1 μM Cd^2+^	3.0 nM Cd^2+^	[72]
AgNPs/biopolymer xylan	River water samples	10–500 μM Hg^2+^	4.6 nM Hg^2+^	[73]
PAA/AgNCs	Tap water and mineral water samples	0–20 μM Hg^2+^	2 nM Hg^2+^	[74]
Carr/Ag/AgCl NPs	Environmental or biological samples	1–100 μM Hg^2+^	1 μM Hg^2+^	[75]
AgNPs	Metal ions sample	3–30 nM Hg^2+^	0.037 nM Hg^2+^	[76]

Abbreviations: 4-mercaptophenylboronic acid (MPBA), Au nanoparticles (AuNPs), ethylenediamine (en), thiosulfate (S_2_O_3_^2^^−^), 4-mercaptobutanol (4-MB), dithioerythritol-modified AuNPs (DTET-AuNPs), graphite-like nitride doped carbon quantum dots (g-CQDs), graphene oxide (GO), thiomalic acid functionalized silver nanoparticles (TMA/AgNPs), sulfosalicylic acid functionalized silver nanoparticles (SAA/AgNPs), poly(acrylic acid)-templated silver nanoclusters (PAA/AgNCs), carrageenan (Carr), Ag nanoparticles (AgNPs).

### 2.3. Fluorescent Analysis by Nanofluorophores and Nanoquenchers

#### 2.3.1. Quantum Dots

Fluorescence resonance energy transfer (FRET) is an optical phenomenon in which energy is transferred from the donor fluorophore to the recipient fluorophore through a non-radiation path. In recent years, efforts have been made to develop nanomaterials into FRET sensors (Table 4). Traditional semiconductor quantum dots (QDs) are particles usually below 10 nm in size. The exceptionally high surface-to-volume ratio brings excellent optical properties. QDs far outweigh organic fluorophores in fluorescence intensity, excitation and emission ranges, and photostability [77].

The fluorescence of cadmium telluride (CdTe) QDs capped by mercaptopropionic acid (MPA) can be quenched in EDTA system and then selectively restored by Zn^2+^. The “turned on” QD sensor has been applied to the detection of trace Zn^2+^ in zinc-fortified salt, energy drinks, and different foods [78]. Fluorescence of QDs can also be “turned off” in the presence of heavy metal ions. Citric acid-capped cadmium sulfide (CdS) QDs can selectively interact with Cu^2+^ and thus reducing the fluorescence intensity; such property of CdS/QDs has been applied in analyzing environmental samples [79]. The fluorescence emission of (E)-2,2′-(4,4′-dioxo-2,2′-dithioxo-2H,2′H-[5,5′-bithiazolylidene]-3,3′(4H,4′H)-diyl) bis(3-mercaptopropanoic acid) (DTM)-capped CdTe/CdS QDs can be reduced by the strong and stable Hg^2+^/DTM complex; the selective sensor has been reported for rapid detection of Hg^2+^ in tap water [80]. Compared to intrinsic QDs, transition metal ion-modified QDs are equipped with enhanced thermal stability, reduced chemical sensitivity, and eliminated self-quenching and re-absorption due to large Stokes displacement [81]. For example, Mn-modified ZnSe/ZnS QDs were used for Hg^2+^ detection, respectively, based on selective fluorescence quenching of heavy metal ions [82].

Carbon quantum dots (CQDs) exhibit stronger fluorescence properties and lower toxicity than traditional QDs. Nitrogen and sulfur co-doped CQDs (N, S-CDs) exhibits an improved fluorescence quantum yield (QY) [83] and excitation-independent emission [84]. Fluorescence of N, S-CQDs can be “turned off” by Cr(VI) and Hg^2+^, respectively. Because of higher affinity between Hg^2+^ and I^−^, the Hg^2+^-CQDs system can be further “turned on” by I^−^. Therefore, such CQDs have been successfully used to detect Cr(VI) [85], Hg^2+^, and I^−^ [86] in food and water samples. However, the application of CQDs-based fluorescence sensor in field detection has been limited, since CQDs usually need to be mixed with water samples. To overcome such drawbacks, much effort has been devoted to immobilizing CQDs in a hydrogel matrix with integrated photonic functions. Photonic devices composed of CQDs-doped hydrogel waveguides can be directly used to detect Hg^2+^ in water samples [87]. Furthermore, graphene quantum dots can remain fluorescence for 4–5 months; they have been successfully used for selective recognition of Pb^2+^ and Cr^3+^ in aqueous media [88].

#### 2.3.2. Gold Nanoclusters

Metal nanoclusters (NCs) possess several advantages as promising fluorescent probes: low toxicity, high photoluminescence, and good biocompatibility [89]. Noble metal NCs usually compose of several to dozens of atoms; their size are less than 1 nm which is comparable to the Fermi wavelength of conducting electrons. The spatial limitation of free electrons in metal NCs results in discrete and size adjustable electronic transitions, contributing to molecular-like properties, such as luminescence and unique charging characteristics [90].

**Figure 4:**
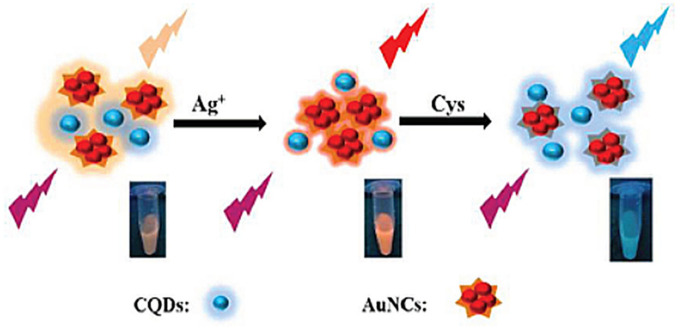
Schematic illustration of the detection of Ag^+^ and L-cysteine with dual emissive nano system of CQDs and AuNCs [93]. Reproduced with permission from Han et al., *Anal. Method.*; published by The Royal Society of Chemistry, 2014.

Fluorescent AuNCs usually consists of an inorganic gold core and an organic ligand shell [91]; the core–shell structure can be modified in multiple ways in order to construct multi-functional optical chemical/biological sensors [92]. Since CQDs and AuNCs can be excited by a single wavelength simultaneously, a dual-emission nano system of CDs and AuNCs was constructed for real-time visual detection of Ag^+^ in the presence of other environmentally relevant metal ions (Figure 4) [93]. A double-emission ratio fluorescent nanoprobe (RFN) was synthesized in three steps: (1) doping SiO_2_ with fluorescein isothiocyanate (FITC), (2) coating the FITC-SiO_2_ microspheres with amino groups, and (3) combining AuNCs on the surface via interaction with amino groups. The RFN successfully determined trace Hg^2+^ in environmental water samples, and showed prospect in monitoring heavy metal ions, pollutants and environmental protection [94]. Another RFN was synthesized by covalently linking 11-mercaptoundecanoic acid-stabilized AuNCs to the surface of amino-functionalized CD/SiO_2_ nanoparticles. The sensor has successfully achieved sensitive and accurate visual detection of Cu^2+^ in water samples and human serum samples, showing promises in environmental monitoring and medical diagnosis [95].

#### 2.3.3. Graphene and Its Derivatives

Graphene and its derivatives can not only act as energy receptors to quench various fluorophores but also as fluorescence donors to produce fluorescence signals under certain circumstances. AuNPs-functionalized graphene can be used as an effective fluorescent sensing platform for Pb^2+^ in environmental water samples. The sensing platform shows minimal background fluorescence because AuNPs have extremely high quenching capacity. With the addition of Pb^2+^, the fluorescence restoration was enhanced significantly by accelerating the extraction rate of AuNPs in the presence of thiosulphates and 2-mercaptoethanol on graphene surface [96].

GO has a large number of carboxyl groups, hydroxyl groups, and epoxy groups, which usually lead to non-emission and non-radiative recombination of local electron hole pairs. After modification of these oxygen-containing groups, the non-radiative composite sites can be removed, and the photoluminescence properties of the modified GO can be exhibited [97,98]. A GO-based fluorescence nano sensor could be designed by covalent grafting of allylamine on the surface of GO. In aqueous media, the sensor displays a highly selective and sensitive discrimination of Fe^3+^ from Fe^2+^ and other metal ions through electron transfer-induced fluorescence quenching [99]. 8-hydroxyquinoline (8-HQ) has good binding ability to many divalent and trivalent metal ions [100].8-HQ-functionalized GO has been developed for the fluorescence detection of Zn^2+^: fluorescent emission of the nanosensor can be “turned on” at 366 nm, which could be attributed to chelating mode II of 8-HQ binding to Zn^2+^, forming complex near the GO sheet. GO-8-HQ exhibits satisfactory selectivity and sensitivity toward Zn^2+^ ions in aqueous media [101].

In addition, graphitic carbon nitride nanosheets (GCNNs) is a 2D material with unique and excellent physical and chemical properties [102]. High fluorescence GCNNs were reported prepared via an environmentally friendly one-step synthetic pathway, using sodium citrate and melamine as carbon and nitrogen sources. Fluorescence of such sensor can be selectively quenched by Hg^2+^; it has been successfully used for the detection of Hg^2+^ in water and milk samples [103].

**Table 4:** Representative studies of fluorescent analysis of heavy metals from food and water resources by nanofluorophores and nanoquenchers.

Electrode	Sample	Linear Range	Limits of Detectio	Ref.
EDTA/(CdTe) QDs/MPA	Zinc fortified table salts and energy drinks sample	9.1–109.1 μM Zn^2+^	2 μM Zn^2+^	[78]
CA-CdS QDs	Real water sample	10–5000 nM Cu^2+^	9.2 nM Cu^2+^	[79]
DTM/(CdTe/CdS) QDs	Tap water sample	0.3–21 nM Hg^2+^	0.08 nM Hg^2+^	[80]
Mn/QDs (ZnSe/ZnS)/MPA	Real drinking water	0–20 nM Hg^2+^	0.1 nM Hg^2+^	[82]
S, N-CQDs	Tap water sample	0.065–198 μmol/L Cr(VI)	0.56 nmol/L Cr(VI)	[85]
N, S-CQDs	Tap, river and mineral waters and fish samples.	0.1–20 μM Hg^2+^ 0.1–10 μM I^−^	62 nM Hg^2+^ 72 nM I^−^	[86]
hydrogel/CQDs	Water samples	0–5 μM Hg^2+^	4 nM Hg^2+^	[87]
GQD	Water sample	/	100 nM Cr^3+^ 100 nm Pb^2+^	[88]
CQDs/AuNCs	Aqueous solution	10–1000 nM Ag^+^	2 nM Ag^+^	[93]
FITC–SiO_2_-amino groups -AuNCs	Water samples	0.1–10 nM Hg^2+^	0.1 nM Hg^2+^	[94]
11-MUA-AuNCs-CQDs-SiO_2_	Real water samples and human serum samples	0.025–4 μM Cu^2+^	0.013 μM Cu^2+^	[95]
S_2_O_3_^2−^/2-ME/AuNPs/G	Tap water and mineral water samples	50–1000 nM Pb^2+^	10 nM Pb^2+^	[96]
GO	Aqueous solution	0–120 μM Fe^3+^	4.6 μM Fe^3+^	[99]
GO/8-HQ	Aqueous solution	0.05-1.5 μM (Zn^2+^)	/	[101]
GCNNs	Water and milk samples	0.001–1.0 μM Hg^2+^	0.3 nM Hg^2+^	[103]

Abbreviations: Cadmium telluride (CdTe), quantum dots (QDs), mercaptopropionic acid (MPA), citric acid-capped cadmium sulfide quantum dots (CA-CdS QDs), (E)-2,2′-(4,4′-dioxo-2,2′-dithioxo-2H,2′H-[5,5′-bithiazolylidene]-3,3′(4H,4′H)-diyl) bis(3-mercaptopropanoic acid) (DTM), mercaptopropionic acid (MPA), sulfur and nitrogen dual-doped carbon quantum dots (S, N-CQDs), carbon quantum dots (CQDs), graphene quantum dots (GQD), fluoresce isothiocyanate (FITC), 11-mercaptoundecanoic acid (11-MUA), AuNPs on graphene surfaces in the presence of both thiosulfate (S_2_O_3_^2−^) and 2-mercaptoethanol (2-ME) (S_2_O_3_^2−^/2-ME/AuNPs/G), graphene oxide (GO), 8-hydroxyquinoline (8-HQ), graphitic carbon nitrides nanosheets (GCNNs).

### 2.4. Nanomaterial-Improved Biosensing

#### 2.4.1. Gold and Silver Nanoparticles

In a biosensor, an incorporated biological component (e.g. nucleotides, enzymes, antibodies) specifically interacts with its target analytes; the generated biochemical changes are usually transformed into electrochemical or colorimetric signals, which can be detected by a nanomaterial-based sensing component [104,105] (Table 5). Among various type of biological components used in biosensors, aptamers are single stranded oligonucleotides, which can be synthesized to bind target molecules with high affinity and specificity [106,107]. Compared with other biological materials, aptamers exhibit outstanding resistance of high temperature, extreme pH values, and harsh chemical conditions [108]; they have a wide range of targets including inorganic/small organic molecules, peptides/proteins, carbohydrates, antibiotics, as well as cells and organisms [109]. Synthesized aptamers can be selected via a process called Systematic Evolution of Ligands by Exponential Enrichment (SELEX) [110,111], where the G-quartets and G-quadruplexes of the selected nucleotide chain intrinsically have specific interactions with multiple metal ions, including K^+^, Hg^2+^, and Pb^2+^ [112].

**Figure 5:**
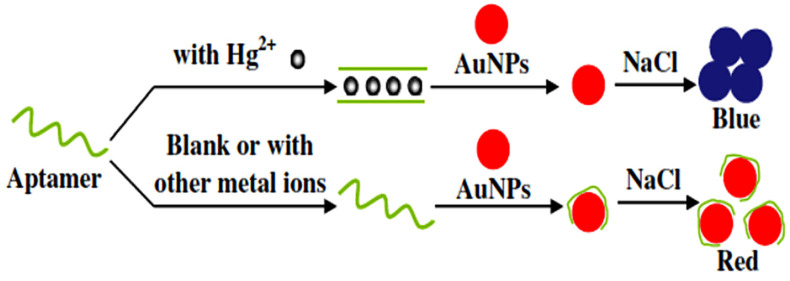
Schematic illustration of the AuNP colorimetric strategy for Hg^2+^ detection [115]. Reproduced with permission from Li et al., *Anal. Bioanal. Chem.* published by the Springer-Verlag 2009.

AuNPs have excellent catalytic properties, electrical conductivity, and biocompatibility with enzymes. They can be widely used in electrochemical biosensors to improve their response, stability, and sensitivity. T–Hg^2+^–T coordination technology can be used to detect Hg^2+^ [113]. Aptamer and target binding results in the desorption of aptamer from the surface of AuNPs, while the residual aptamer adsorbed on the surface of AuNPs triggers the growth of AuNPs with different morphologies and nanostructures, forming solutions in different colors. Bare AuNPs interact with short single-stranded DNA (ssDNA) and double-stranded DNA (dsDNA) in different ways. In the presence of Hg^2+^, two single-stranded oligonucleotides can easily form DNA double stranded structure through T–Hg^2+^–T complex. Double- and single-stranded oligonucleotides have different electrostatic properties. The essential difference is that ssDNA can dissociate sufficiently to expose their bases, while dsDNA have stable double helix geometry and always present a negatively charged phosphoric acid backbone. Therefore, a short ssDNA (usually shorter than 30 bases) can be quickly adsorbed on the bare colloidal gold nanoparticles, while a long ssDNA and dsDNA cannot [114], which indicates that only ssDNA can be used. 8-mer was used to detect Hg^2+^ with improved sensitivity. The sensor has been successfully applied in the determination of Hg^2+^ in tap water and lake water samples [115] (Figure 5).

AuNPs are also commonly used as a tracer in lateral flow strip (LFS). However, most of the LFSs based on AuNPs are not sensitive to heavy metals. In contrast, fluorescent LFS has a higher sensitivity and accuracy. Latex fluorescent microspheres (FMs) are a type of tracers used in fluorescent LFSs [116]. FMs coupled antigen was coated on test-line to provide a fluorescence signal. With the use of AuNPs coupling antibody as quenching agent, detection of Hg^2+^ can be carried out based on T–Hg^2+^–T principle, which is mentioned above. This method can detect Hg^2+^ in river water samples sensitively and quickly [117].

AuNPs can be widely used in electrochemical biosensors, AuNPs enhance the electron transfer reactions and improve the analytical performance of the biosensors [118]. Based on chitosan (CS), AuNPs modified GCE and aptamer/(AuNPs/CS)_2_/GCE, an aptamer electrochemical sensor with high sensitivity and selectivity was developed. CS was used to immobilize AuNPs on electrodes, while AuNPs were used to immobilize aptamers and enhance electron transfer to improve analytical response. The result shows that the aptamer biosensor is a promising method for trace Hg^2+^ detection in real samples and it has a good practical application prospect. [119]

Similar to AuNPs, AgNPs can also be linked to biomolecules. Based on reduced glutathione (GSH)-stabilized AgNPs in the presence of L-cysteine (Cys), a biosensor for the detection of Al^3+^ was developed via induced aggregation. GSH molecules is a stabilizer which can be adsorbed on AgNPs surface by thiol group (-SH). However, Cys (another small molecule containing thiol group) may attack the surface of AgNPs to some extent and replace GSH, resulting in destabilization and aggregation of AgNPs. In addition, due to the unique and strong interaction between thiol group and AgNPs, Cys can etch AgNPs [120], thus increasing the size of nanoparticles. Since Al^3+^ can form a complex with GSH [121], the Cys aggregates etch the smaller GSH-AgNPs to a large extent after incubation with Al^3+^. The serious aggregation of the GSH-AgNPs results in the obvious color change from yellow to red brown, as well as the red shift and a reduced intensity in LSPRs absorption. It shows that the method can be used for the detection of real water samples (tap water sample) [122]. In addition, sodium salt of N-cholyl-L-cysteine (NaCysC) can be used as reducing agent and stabilizer to synthesize AgNPs through size and shape control. Results reported in the literature show that only Hg^2+^ can trigger the aggregation of smaller corrosion AgNPs, while the other metal ions or anions have no obvious influence on the color and UV-Vis spectra of GSH-AgNPs dispersions. Therefore, the sensor is suitable for the detection of Hg^2+^ in real environment samples (tap water and drinking water) [123,124].

#### 2.4.2. Quantum Dots

QDs have been widely explored in biolabeling. Among all semiconductors, CdS QDs provide a wide spectral band gap tunability. They has been modified with various surfactants, biomolecules, and organic molecules that act as capping agents and fluorescence tools for detecting discrete metal ions in aqueous media [125,126]. GSH is a thiol containing important biological oligopeptide, which composed of active carboxyl and amino groups. It can be chelated with Cd ions to generate QDs. The synthetic CdS QDs are environmentally friendly and biocompatibility that can be used in sensing applications. The fluorescent probe is suitable for the detection in real samples (tap water and lake water sample) [127].

Luminescent CdSe semiconductor QDs clad denatured bovine serum albumin (dBSA) shells were used for copper (II) ion detection. Cu^2+^ ions were reduced to Cu^+^ through the dBSA shell, and then Cu^+^ and Cd^2+^ were chemically replaced on the surface in the ultra-micro-small metal core, thus affecting the fluorescence performance. Furthermore, the dBSA capping can avail a numerous functional biomass or biomolecules to facilitate further modifications for biocompatible QD synthesis. QDs have the potential to be developed into a fluorescent probe for the detection of heavy metals in aqueous solution [128].

In recent years, a lot of effort has been put into the biosynthesis of QDs for developing environment-friendly process, mild reaction conditions, and good biocompatibility [129,130]. Biosynthesis is achieved by simply adding the appropriate enzymes and/or reaction precursors, rather than using entire microorganisms based on a full understanding of the enzymes involved in some substrates. A common sulfur dual element CdSe QDs was synthesized in *Bacillus licheniformis* (*B. licheniformis*) and used as an integral biological probe for detection without further separation. As a nanomaterial for intracellular biosynthesis, QDs are naturally protected by bacterial semi-permeable membrane, which can selectively transport molecules. Cu^2+^ can be detected without additional purification and modification. It is a selective, reliable, and simple method for monitoring and screening of Cu^2+^ in complex environmental samples [131].

#### 2.4.3. Magnetic Nanoparticles

Sensors that combine magnetic nanoparticles and biomolecules can be used to detect heavy metals. Most of them consist of electrochemical detectors. Fe_3_O_4_ NPs is one of the most widely used magnetic nanomaterials with high surface volume ratio and magnetic properties. Fe_3_O_4_ has attracted much attention due to its novel magnetic and catalytic properties. Fe_3_O_4_-based composites can be used as a platform for DNA biosensors. DNA modified Fe_3_O_4_/AuNPs, and magnetic glassy carbon electrode were used to detect Ag^+^ and Hg^2+^ with high sensitivity and selectivity [132]. DNA probe (DNA1) was thiolated on the Fe_3_O_4_/AuNPs surface. The DNA probes (DNA 2 and 3), labeled with two electrochemical substances consisting of ferrocene and methylene blue, could characterize the levels of different types of heavy metal ions simultaneously. The target was identified by using stable metal ions coordination DNA base pairs on the surface Fe_3_O_4_/AuNPs. The sensing strategy designs specific interactions between metal ions and mismatched base pairs (C-C and T-T). Specifically, the stable structure of cytosine-Ag^+^-cytosine and thymine-Hg^2+^-thymine formed in the presence of Ag^+^ and Hg^2+^ can assist the hybridization of DNA1/DNA2 and DNA1/DNA3, and corresponding electrochemical substances can be found on the magnetic Fe_3_O_4_/AuNPs surface. The method has been successfully applied to the analysis of Ag^+^ and Hg^2+^ in lake water, drinking water, orange juice, and red wine, and it has a potential application for monitoring various water samples [133].

Besides, the MOFs can be used as a biosensor for the identification of small heavy metal ions or nucleic acid molecules. It is because the special functional groups of organic ligands in MOF provide the source of stacking, hydrogen bonding, and electrostatic interactions with negatively charged nucleic acid sequences [134,135]. Among all kinds of MOF materials, as an electrode material, Fe^3+^ based MOF (Fe-MOF) has a good stability and redox activity. A new core–shell nanostructured composite of Fe-MOF and m Fe_3_O_4_/mC (denoted as Fe-MOF/mFe_3_O_4_/mC) possesses the inner cavity and orderly mesoporous opening structure. Then the aptamer strands were fixed and the heavy metal ions (Pb^2+^ and As^3+^) were detected through the biometric interaction between the aptamer strands and heavy metal ions. Due to supramolecular stacking and hydrogen bond interaction, the tight binding between Fe-MOF and aptamer chain can produce a higher fixation force for aptamer chain. The nanocomposite material can be applied to detect Pb^2+^ and As^3+^ in the river water and the human blood serum samples [136].

Porphyrin ligands are a class of ligands with wide applications. These porphyrin derivatives play a key role in many chemical and biological processes. When MOF is used as a platform for immobilizing porphyrin groups, its rigid structure with high specific surface area and porosity can not only make each porphyrin contact with the substrate, but also prevent the dimerization of reaction center, thus blocking the catalytic pathway [137]. Therefore, DNA (GR) functionalized iron porphyrin metal organic framework (Fe-P)n-MOF-Au-GR) was used as a signal probe, while a novel gold nanoparticle modified paper electrode (Au-PWE) was used as a high sensitivity electrochemical biosensor. In the presence of Pb^2+^, GR could cleave specifically at the ribonucleotide (rA) site to produce a short (Fe-P)n-MOF linked oligonucleotide fragment and hybridize with the hairpin DNA fixed on the surface of Au-PWE. As (Fe-P)n-MOF has properties similar to peroxidase, the electrochemical signal is amplified by the enzyme, which allows the sensitive detection of Pb^2+^. In addition, Pb^2+^ dependent GR can selectively detect Pb^2+^ in the presence of other metal ions. The method can be applied to water samples (industrial wastewater samples and river water sample), fruit juice samples (orange juice and apple juice), solid samples and other real samples (real sample preparation in supporting data) [138].

#### 2.4.4. Graphene and Its Derivatives

Graphene DNA biosensors can establish a platform for biological cognition. Most graphene DNA biosensors are developed based on the adsorption between nucleotides and graphene surface, or condensation reaction between the amino group of amino modified DNA and the carboxyl group of GO [139,140]. However, there are some problems in these methods, we should develop new binding methods of DNA and graphene. Dopamine was selected as reducing agent to prepare dopamine-functionalized rGO, and NH_2_-ssDNA was grafted onto its surface. In the absence of Hg^2+^, the target DNA of four thymine–thymine (T-T) mismatches could not be hybridized with probe DNA on the glassy carbon electrode, but they can be hybridized by T-Hg^2+^-T coordination chemistry in the presence of Hg^2+^. This method has been successfully applied to the quick, convenient, and effective detection of Hg^2+^ in environmental samples [141].

GO are coupled with the aptamer functionalized CdSe/ZnS QDs to form a “turn-on” fluorescent sensor for detecting Pb^2+^. QDs (CdSe/ZnS) acts as the energy donor and GO serves as the energy acceptor. In the presence of Pb^2+^, the interaction between Pb^2+^ and the aptamer resulted in the conformational change of the aptamer, forming the G-quadruplex/Pb^2+^ complex. As a result, the QDs linked to the G-quadruplex/Pb^2+^ complex was separated from the GO sheet, which “turn-on” the fluorescence of QDs. The sensor can be applied to the detection of Pb^2+^ in river water samples [142]. Since GO can act as an indicator of electrochemical signals, Hg^2+^ ions can be detected by measuring the current of GO during electrochemical reduction. Thiol-functionalized poly-T-oligonucleotides were used as a platform for sensing Hg^2+^ ions on the gold electrode surface. T-Hg^2+^-T bonds were formed by inserting Hg^2+^ ions between T-T mismatches, and the π-π interaction between ssDNA and GO was stronger than that of dsDNA [143,144], therefore, the structure of the DNA transitioned from a single-stranded to a double-stranded form. The current required for electrochemical reduction of GO was monitored on the generated surface, showing a linear response in the range of 1-300 nm Hg^2+^ ion concentration, and has high selectivity for Hg^2+^ ions [145].

#### 2.4.5. Other Materials

Up conversion nanoparticles (UCNPs) as fluorescent biomarkers have attracted great attentions from researchers due to their attractive optical and chemical properties, including low toxicity, large Stokes’s shift, high resistance to photobleaching, scintillation, and photochemical stability [146]. Lanthanide-based UCNPs can emit short wavelength in different colors under the excitation of near infrared light [147]. The sensitivity can be improved to a certain extent due to the absence of autofluorescence. A turn-on nano sensor was developed based on FRET between long-strand aptamers functionalized UCNPs and short-strand aptamers functionalized AuNPs. In the absence of Hg^2+^, FRET between UCNPs and AuNPs occurred owing to the specific matching between two aptamers, resulting in the fluorescence quenching of UCNPs. The method can be used for the determination of Hg^2+^ in tap water and milk samples with good accuracy and selectivity [148].

CQDs contain a variety of functional groups, including carboxyl and amino groups. Obviously, CQDs, as a polydentate ligand, has a good application prospect in the construction of new optical sensors using AuNPs. Based on CQDs induced aggregation of AuNPs assisted by GSH, Hg^2+^ ions were detected by colorimetry. In this sensor system, AuNPs/CQDs composite is formed by Au-N bond. At the same time, the color of the solution changed from wine red to blue. When GSH is added, the competitive binding of GSH and CQDs on the surface of AuNP can protect AuNP from aggregation, thus the well dispersed AuNPs can be recovered. In the presence of Hg^2+^ ions, GSH can chelate with Hg^2+^ to form a complex, which leading to CQDs induced aggregation, thus achieving the detection of Hg^2+^ [149].

**Table 5:** Representative studies of probed-based detection of heavy metals from food and water resources by nanosensors.

Electrode	Analytical Technique	Sample	Linear Range	Limits of Detection	Aptamer Sequence	Ref.
AuNPs-aptamer	Colorimetry	Water samples	/1 nM–0.1 mM Hg^2+^	0.6 nM Hg^2+^	5′-TTTTTTTTTT-3′(Hg^2+^); 5′-CCAACCACAC-3′ (Control random oligonucleotide sequence)	[115]
FMs/AuNPs-aptamer	Fluorometry	River water samples	0.13–4 ng/mL Hg^2+^	0.13 ng/mL Hg^2+^	MRP: 5′-biotin-AAA AAA AAA ATT CTT TCT TCC CCT TGT TTG TT-3′; T-line Probe1: 5′-biotin-AAA AAA AAA ACA CAA ACA AGG CCA ACA-3′	[117]
(AuNPs/CS)_2_-GCE- aptamer	Electrochemical	Tap water samples	0.01–500 nM Hg^2+^	0.005 nM Hg^2+^	5′-HS-(CH_2_)_6_-TCA TGT TTG TTT GTT GGC CCC CCT TCT TTC TTA-Fc-3′	[119]
GSH/AgNPs (Cys)	Colorimetry	Water samples	0.4–4.0 μM Al^3+^	1.2 μM (eyes) Al^3+^ 0.16 μM (UV) Al^3+^	/	[122]
AgNPs/NaCysC	Colorimetry	Tap water and drinking water	5–50 nM Hg^2+^	8 nM Hg^2+^	/	[124]
CdS (QDs)/GSH	Fluorometry	Industrial wastewater sample	10 nM–20 μM Cd^2+^	0.54 nM Cd^2+^	/	[127]
CdSe (QDs) Bacillus licheniformis cells	Fluorometry	Complex solution environment	0–20 μM Cu^2+^	0.91 μM Cu^2+^	/	[130]
DNA/Fe_3_O_4_/AuNPs/MGCE	Electrochemical	Taihu Lake water, drinking water, orange juice and red wine sample	10–150 nM Ag^+^ 10–100 nM Hg^2+^	3.4 nM Ag^+^ 1.7 nM Hg^2+^	DNA1 (thiolate), DNA2 (Fc and MB)	[133]
Fe-MOF/mFe_3_O_4_mC-aptamer	Electrochemical	River water samples	0.01–10.0 nM Pb^2+^	2.2 pM Pb^2+^	5′-CAA-CGG-TGG-GTG-TGG-TTGG-3′	[136]
Fe-MOF/mFe_3_O_4_mC-aptamer	Electrochemical	River water samples	0.01–10.0 nM As^3+^	6.73 pM As^3+^	5′-GGT-AAT-ACG-ACT-CAC-TAT-AGG-GAG-ATA-CCA-GCT-TAT-TCAATT-TTA-CAG-AAC -AAC-CAA-CGT-CGC-TCC-GGG-TAC-TTC-TTC-ATC-GAG-ATA-GTAAGT-GCA-ATCT-3′	[136]
Au-PWE/((Fe-P)n-MOF-Au-GR)	Electrochemical	Water, fruit juice soil sample	0.03–1000 Pb^2+^	0.02 Pb^2+^	/	[138]
PDA/rGO/DNA/GCE	Electrochemical	Kunyu River sample	8–100 nM Hg^2+^	5 nM Hg^2+^	Probe DNA (NH^2−^ssDNA: 5′-NH^2−^(CH_2_)_6_-GAT-TCC-GTG-CAT-GAC-TCA-G-3′) Target DNA (4-Mis DNA:5′-C-TGT-GTC-TTG-CTC-GGT-ATC-3′) Control DNA (5′-GAT-TCC-GTG-CAT-GAC-TCA-G-3′)	[141]
GO/aptamer- CdSe/ZnS (QDs)	Fluorometry	River water samples	0.1–10 nM Pb^2+^	90 pM Pb^2+^	5′-NH_2_^−^(CH_2_)_6_–GGGTGGGTGGGTGGGT–3′	[142]
GO/Au electrode (Thiol-PTO)	Electrochemical	Drinking water	1–300 nM Hg^2+^	1 nM Hg^2+^	(Thiol-PTO) (SH-C_6_-5′TTT-TTT-TTT-TTT-TTT-TTT-TTT-TTT-TTT-TTT-3′)	[145]
Long strand aptamer UCNPs/AuNPs-Short strand aptamer	Fluorometry	Tap water and milk samples	0.2–20 μM Hg^2+^	60 nM Hg^2+^	5’NH_2_C_6_-CTA CAG TTT CAC CTT TTC CCC CGT TTT GGT GTT T-3′ (Long stranded aptamer), (short-stranded aptamer) 5’SHC_6_-GAA ACT GTA G-3’	[148]
CQDs/AuNPs/GSH	Colorimetry	Environmental Water samples	10–300 nM Hg^2+^	7.5 nM Hg^2+^	/	[149]

Abbreviations: Au nanoparticles (AuNPs), latex fluorescent microspheres (FMs), chitosan (CS), glassy carbon electrode (GCE), glutathione (GSH), L-cysteine (Cys), Sodium salt of N-cholyl-L-cysteine (NaCysC), cadmium sulfide (CdS), magnetic glassy carbon electrode (MGCE), Fe^3+^-based metal–organic framework (Fe-MOF), DNA (GR) functionalized iron porphyrin metal organic framework (Fe-P)n-MOF-Au-GR), gold nanoparticle modified paper electrode (Au-PWE), polydopamine (PDA), quantum dots (QDs), poly-T-oligonucleotides (PTO), up-conversion nanoparticles (UCNPs).

## 3. Nanotechnology-Based Heavy Metal Removal in Food and Water

Although the use of commercially available activated carbon and different grades of zeolite is still popular, these processes are very expensive [150]. In recent years, green chemical methods including natural biopolymers and biological wastes have been developed to synthesize magnetic nano adsorbents due to low cost, high availability, biodegradability and strong affinity for metal capping. As a kind of nano material, magnetic nanoparticles have a large surface area and high adsorption capacity, thus improving the purification efficiency. Unfortunately, they are unstable in acid or alkaline conditions, resulting in reduced magnetic force and shorter life. To overcome the drawbacks, chemical modification on the surface of adsorbent is needed for the effective removal of heavy metal ions [151]. The removal of heavy metals by metal oxide nanoparticles, MNPs, graphene, its oxides and its derivatives, CNTs, and other nanomaterial will be discussed in the following sections (Table 6).

### 3.1. Metal Oxide Nanoparticles

Due to their unique physical and chemical properties, metal oxide nanoparticles have been employed in the removal of toxic heavy metal ions from polluted water. In recent years, green chemical methods including natural biopolymers and biological wastes have been developed to synthesize magnetic nano adsorbents due to low cost, high availability, biodegradability, and strong affinity for metal capping.

The removal of chromium was carried out by adsorption, with the use of Mo(IV) as the active site and accompanied with redox reaction in the removal process [152]. Molybdenum disulfide (MoS_2_) is a typical layered transition metal dichloride [153]. According to the spatial arrangement between Mo and S atoms, MoS_2_ can be divided into two different crystal phases: semiconductor 2H and metastable 1T [154]. Metalic1T phase has a completely different electronic structure. Compared with the semiconductor 2H phase, MoS_2_ shows higher chemical activity because of the lower charge transfer resistance, different electronic properties and larger interlayer spacing in 1T phase [155,156]. The ultrasTtable 1T-MoS_2_ with wide interlayer space was synthesized in the presence of water and ethanol. The formation of sulfur vacancies on M-MoS_2_ by ethanol induced solvothermal reaction accelerated the conversion of 2H to 1T phase in MoS_2_. Meantime, the strong interaction between ethanol and MoS_2_ surface reduces the total energy of MoS_2_ nanosheets, which improves the stability of 1T-MoS_2_. The ultrastable 1T- MoS_2_ is an ideal material for heavy metal ion purification; however, it is difficult to synthesize high purity 1T-MoS_2_ directly because of its high formation energy. MoS_2_ can be used as a new adsorption material for Cr(VI) removal, with good adsorption capacity, stability and recyclability.

In addition, CuO with different structures was synthesized at the nano scale, which showed good adsorption properties for As^3+^, As(V), Pb^2+^, and Cr(VI). CuO nanoparticles with high specific surface area and uniform particle size distribution can be prepared by cold finger assisted magnetron sputtering technique, which has an important adsorption effect on the removal of heavy metal ions [157]. The adsorption ability can be improved by increasing the adsorption site and surface area of CuO. The structure modification of CuO at nanoscale enhances the number of active centers for metal ions, which plays a key role in removing heavy metal ions.

The preparation of SiO_2_ mesoporous nanoparticles with organic surfactants is a new way to solve the environmental problems caused by Cr^3+^ ion. Organo-silica mesoporous materials with cyano functional groups were prepared by a one pot co-condensation of 2-cyanoethyltriethoxysilane and tetraethoxysilane at ratios of 1:4 and 1:9 using either sunflower oil or n-dodecyl amine as templating agents. Cyano group was used as adsorption site or hydrolyzed to carboxyl surface functional group. The removal rate of Cr^3+^ ion is 48–83%, which depends on the functional groups of adsorbents and the ratio of silicone to silica. The results indicated that the prepared material is a good adsorbent [158].

### 3.2. Magnetic Nanoparticles

Magnetic Fe_3_O_4_ nanoparticles are often modified with a surface coating to (1) avoid aggregation and oxidation and (2) increase selectivity to specific targets. For example, a core–shell structure was reported consisting of a core of Fe_3_O_4_, SiO_2_, and polythiophene. It can be used for rapid separation and enrichment of Hg^2+^ ions in various matrices [159]. Another example, Fe_3_O_4_ hybridized with polyaniline and MnO_2_ (Fe_3_O_4_/PANI/MnO_2_), can be synthesized economically and in a green way [160,161], and exhibit the strong adsorption capacity for heavy metal ions (including Cd^2+^, Zn^2+^, Pb^2+^, and Cu^2+^) [162]. As a typical amino conjugated polymer, poly-(m-phenylenediamine) (PmPD) is densely packed with amine and imine functional groups, which provide it with excellent redox performance, chelating capacity and a large number of adsorption sites for pollutants [163]. Since PmPD has a similar structure and property to PANI. Therefore, it is feasible to modify PmPD by MnO_2_ in theory. MnO_2_/Fe_3_O_4_/PmPD core–shell hybrid has a high adsorption ability and inherent paramagnetic property, which can adsorb metal ions through electrostatic attraction, ion exchange, and coordination interaction, facilitating easy separation and removal of heavy metal pollutants at large scale [164].

PPy, as previously described, is a conductive polymer for the removal of hexavalent chromium from water by reducing toxic Cr(VI) to less toxic Cr^3+^. Since amine functional groups can be used as the adsorption sites of Cr(VI), promoting the chelation or adsorption of Cr(VI) ions, many studies have reported that the functionalization of PPy can be done by increasing the number of amine functional groups [165,166]. The addition of m-phenyl diamine (mPD) enhanced the absorption of Cr(VI) by increasing the active sites of amines and the surface area of PPy. Then Fe_3_O_4_ nanoparticles were added into PPy-mPD composite, which can improve the adsorption capacity of Cr(VI), simplify the separation of adsorbent and aqueous solution, as well as prevent the oxidation of Fe_3_O_4_ nanoparticles. PPy-mPD/Fe_3_O_4_ nanocomposite is a potential adsorbent for Cr(VI) removal due to its high adsorption capacity and selectivity. It is also a very low-cost super-adsorption material for heavy metal ions [167].

**Figure 6:**
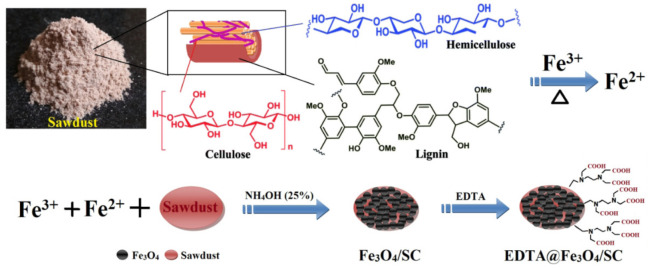
Scheme for synthesis of Fe_3_O_4_/SC and EDTA@Fe_3_O_4_/SC [169]. Reproduced with permission from Kataria et al., *Elsevier B.V.* *Eng.;* published by the chemosphere 2018.

Nano adsorbents were synthesized from rice husk, pine wood, wood, sawdust, orange peel, plant leaves, and other biological wastes. Sawdust readily available, low cost, and which contains lignocellulose, for reduction of Fe^3+^ to Fe^2+^ ions [168]. Sawdust containing phenols and lignocellulose can be used for metal reduction. Fe_3_O_4_/sawdust composites were prepared utilizing single iron source as a precursor and sawdust as a green reducing agent. In addition, EDTA can be used to modify Fe_3_O_4_/sawdust and increase the adsorption capacity of Cd^2+^. The adsorption mechanism includes electron attraction and chemical adsorption (Figure 6) [169].

In addition, nano zero valent iron (NZVI) has good repair ability and low production cost. The ability to adsorb and purify toxic pollutants, especially inorganic ions and heavy metal ions, has been proved [170,171,172]. However, NZVI is oxidized under the exposure of oxygen, air, and water, leading to the reduction in reactivity. Nickel ions are more stable than iron ions which can be employed to reduce the oxidation rate. Using NaBH_4_ as a reducing agent, nanoscale zerovalent nickel was synthesized by a direct and simple grinding reduction method. It can be used as an adsorbent for Cr(VI) [173].

### 3.3. Graphene and Its Derivatives

Graphene has a large theoretical specific surface area (∼2630 m^2^/g), while GO has an extended layered structure and abundant hydrophilic polar groups, such as hydroxyl (–OH) groups, epoxy resin, and carboxyl (–COOH) groups, indicating its potential to remove heavy metal ions and organic pollutants [174,175]. However, due to the small particle size of graphene, it is difficult to separate graphene from aqueous solution by the traditional centrifugal filtration method [176]. Therefore, graphene-based magnetic adsorbents have been applied in the field of environmental remediation because they can be separated easily under the action of magnetic field. A ternary magnetic composite was prepared by rGO, PPy, and Fe_3_O_4_ nanoparticles (PPy/Fe_3_O_4_/rGO). PPy has a positively charged nitrogen atom in the polymer chain, which can adsorb and combine with heavy metals easily. For PPy/Fe_3_O_4_/rGO, it has been demonstrated that the Cr(VI) removal is mainly conducted through electrostatic attraction and ion exchange process. The presence of other ions had little effect on the removal of Cr(VI), but the coexisting SO_4_^2−^ ligands alone inhibited the adsorption of Cr(VI). It could be a promising material for the removal of Cr(VI) from wastewater [177]. PANI-based adsorbents with low cost and high stability can be used for mercury removal. However, PANI have a limited surface area and active sites, which resulted in a poor adsorption capacity and recovery. In order to improve these problems aniline was polymerized in the presence of rGO/Fe_3_O_4_ nanocomposites [178,179]. The removal of Hg^2+^ is mainly due to the complexation of the nitrogen-containing group in PANI with Hg^2+^. The adsorbent can be regenerated well in hydrochloric acid solution at pH = 2, and PANI in PANI/rGO/Fe_3_O_4_ can inhibit iron leaching during acid regeneration [180].

In addition, GO was magnetized by mixing FeCl_3_·6H_2_O, FeCl_2_·4H_2_O, and GO with distilled water. In this way, magnetic graphene oxide can be obtained [181]. Then, magnetic nanoparticles and additional carbon supports were grafted onto a novel cyanogen containing porous silica matrix (MgO/SiO_2_^−^CN). The results show that the overall adsorption performance of composite is good, and its magnetic separation performance is significantly enhanced. The adsorption of lead is achieved by the complexation of Pb^2+^ with grafted -C≡N bond. The synergistic effect of the three components (i.e. magnetic graphene oxide matrix, three bonds containing organic parts and inorganic porous silica framework) greatly improves the adsorption performance, making it an ideal adsorbent for removing Pb^2+^ [182].

### 3.4. Carbon Nanotubes

CNTs are proven to be superior adsorbents for several divalent metal ions in water because of their large surface areas and the capability to establish (π-π) electrostatic interactions [183,184]. However, the poor interaction between carbon nanotubes molecules hinders the dispersion of carbon nanotubes in solvents to a great extent. Chemical functionalization is a common method to improve the dispersion of CNTs and realize their huge properties. The formation of functional groups on the surface of CNTs improves the reaction activity and provides active sites for further chemical modification. Generally speaking, the methods include: (i) amidation or esterification of carboxylated CNTs; and (ii) covalent attachment of functional groups at side wall or original carbon nanotubes.

The adsorption of Hg^2+^ in aqueous solution by unmodified MWCNTs is effective. Under the optimum conditions, the removal rate can reach 100% [185]. The adsorption properties of MWCNTs are mainly related to the functional groups attached on the surface of MWCNTs. In order to improve the adsorption capacity, selectivity and removal efficiency of carbon-based adsorbents and CNTs for heavy metals and organic compounds, CNTs were functionalized with amino and thiol groups. Among these functional groups, thiol have an excellent binding ability with some metals such as Ag^+^, Hg^2+^, Cu^2+^, Ni^2+^, and Zn^2+^. The functionalization of MWCNTs was achieved by reacting ethylenediamine, cyanuric chloride and sodium 2^−^mercaptoethanol in sequence as efficient ways to introduce amine and thiol functional groups onto the nanotube sidewalls. Amino and thiol functionalized MWCNTs were successfully prepared for the removal of Hg^2+^ ions from synthetic and chlor-alkali wastewater [186].

In recent years, some studies on biocompatibility and cytotoxicity of CNTs have been carried out [187,188,189], but it is unclear whether the cytotoxicity is caused by CNT itself or its pollutants, such as metal catalysts or amorphous carbon structure. Since the purification or functionalization of carbon nanotubes can significantly reduce their cytotoxicity and improve their biocompatibility, amino functionalized MWCNTs, rather than the unmodified MWCNTs, were used as an adsorbent to remove Cd^2+^ ions from aqueous solutions. 1,6-hexanediamine, diethylenetriamine, triethylenetetramine, and 1,4-phenylenediamine was applied to functionalize the amino groups of MWCNTs, and the direct coupling of ethylenediamine with carboxylic groups to introduce amino groups via amide formation using *O*-(7-azabenzotriazol-1-yl)-*N*,*N*,*N*’,*N*’-tetramethyluronium hexafluorophosphate was performed. The biocompatibility of ethylenediamine (EDA)-functionalized, oxidized, and control original MWCNT were compared on L929 mouse fibroblast cell line. EDA-functionalized MWCNTs have acceptable biocompatibility in vitro because they do not produce cytotoxicity even at a high concentration. The adsorption capacity and affinity of MWCNTs (untreated and modified) for metal ions mainly depend on the surface groups, pH value, and temperature, rather than the specific surface area, pore volume, and pore diameter [190].

### 3.5. Nanocomposite Films

As the main separation element of membrane-based processes, membranes have attracted great attention from researchers in many water treatment applications such as wastewater treatment, water purification, water disinfection, toxic and non-toxic chemical molecules, and heavy metals [191]. Compared with the original ultrafiltration membrane, nanocomposite materials can increase the surface roughness and the number of pores with decreased pore size, improving the ultrafiltration performance by increasing the selectivity and permeability of the membrane [192].

A new type of GO impregnated with mixed matrix membrane (MMM) and prepared by using polysulfone (PSF) in N, N-dimethylformamide (DMF) for the adsorption of heavy metal ions, such as Cr(VI), Cu^2+^, Pb^2+^, and Cd^2+^. The enhanced solvent-non-solvent porous membranes are more layered, resulting in a higher permeation flux. This membrane can adsorb different heavy metals at natural pH [193]. Similarly, compared with sulfonated pentablock copolymer (s-PBC), the s-PBC/GO nanocomposite membrane has a higher adsorption efficiency as an adsorbent to remove heavy metal ions from aqueous solutions. GO is dispersed in the polymer matrix to maintain the layered superlattice structure and increase the spacing between layers (up to 51 nm); it forms a porous structure, which has a superior adsorption capacity than the polymer itself. The composite membrane can be recovered from the metal ions adsorbed in the membrane after the water purification process [194].

### 3.6. Other Nanomaterials

The MOF is a highly crystalline and porous material with a wide range of applications and can also be used as an adsorption material [195]. An exemplary case of tunable MOFs is the water-stable zirconium-based UiO-66 family which can be functionalized with the strong binding part of heavy metal ions at their inorganic nodes or organic connectors. Inorganic nanoparticles (iNPs), as an adsorbent with high activity and specificity, have been widely studied for heavy metal ion removal [196,197]. It is found that the aggregation of iNPs reduces its adsorption efficiency, but it can be addressed by incorporating the iNPs into the porous matrix of other materials, such as MOF. MOF is an attractive partner of iNPs; it not only prevents the accumulation of iNPs and promotes its recovery after adsorption, but also facilitates its repair ability. In the field of pollutant removal, iNPs have been combined with MOF to produce MOF/iNPs adsorbents with additional functions. MOF/iNPs composite microspheres can remove all kinds of metal ions in water simultaneously, including As^3+^, As(V), Cd^2+^, Cr^3+^, Cr(VI), Cu^2+^, Pb^2+^, and Hg^2+^. It is stable for water purification and can be regenerated by mild acid treatment [198].

Cellulose is the most extensive and renewable biopolymer in nature, and it is a very promising raw material [199]. Banana fiber, which consists of cellulose, hemicellulose, and lignin, is one of the natural phloem fibers which is biodegradable and environmentally friendly that is commonly used in wastewater treatment [200]. Natural cellulose can be classified as a semi crystalline fiber material, which is composed of an amorphous zone and a crystalline zone. As a structural defect, the amorphous region is easily eroded by acid, and then cellulose nanocrystals (CNCs), a single short single crystal nanoparticle, are released. Because of the high specific surface area and many active groups, the modified CNCs exhibit an excellent adsorption property. Nano crystals (CNCs)-graft-n butyl acrylate (CNCs-g-nBA) grafted copolymer composite was synthesized and evaluated for the removal of Pb^2+^ from aqueous solution. The results showed that the cellulose CNCs-g-nBA-grafted copolymer has an effective adsorption capacity for the removal of Pb^2+^ ions from aqueous solutions under optimum conditions [201].

In addition, nanofibers are very suitable for adsorption of heavy metals due to their high porosity, and appropriate mechanical properties [202,203]. The ability of microalgae and macroalgae to adsorb or accumulate heavy metals (such as cadmium, chromium, nickel, lead, zinc, and mercury) from aqueous solutions has been fully demonstrated [204]. Sodium alginate (SA) is a non-toxic, biocompatible, and biodegradable biopolymer extracted from brown algae. It can be used to prepare nanofibers, but modification is needed due to its hard structure, limited solubility and high viscosity. Polyvinyl alcohol (PVA) is a kind of polymer material with high flexibility which can be applied in practical research because of its high film-forming ability and fiber properties [205]. This is due to their water solubility and the formation of stable compounds by strong polymer–polymer and hydrogen bonds [206]. PVA can combine with SA to form SA/PVA nanofibers [207]. Nanocomposite nanofibers were successfully prepared by electrospinning technology to remove Cd^2+^ ions from aqueous solutions [208].

By adding nano adsorbent into polymer matrix in the form of nanocomposite, such as SA, the difficulty of separation of nano adsorbent can be avoided. As mentioned above, SA biopolymers, which consists of blocks of 1–4 linked α-guluronic and β-d-mannuronic acids, can be used to remove toxic metal ions [209]. In the presence of two valent cations, especially Ca^2+^ ions, SA easily forms a cross-linked gel matrix. Alginate (G) consists of carboxyl groups which enable complex formations with metal ions in an aqueous solution, making it an efficient adsorbent [210,211]. Titanate (T) nanotubes have a large surface area and an excellent adsorption capacity. However, they are difficult to separate and recover from the treated solution. This can be solved by adding a nano composite into polymer matrix, such as G, in the form of nanocomposites. Titanate nanotubes were the most promising adsorbents of As^3+^. Compared with T, although the adsorption performance of T/G nanocomposites is weaker, they have an enhanced separation and recovery capacity [212].

**Table 6:** Representative studies of heavy metal removal from food and water resources by nanomaterials.

Nanomaterial	Contact Time	Adsorbed Metal	Removal Efficiency (mg/g)	Ref.
1T-MoS_2_; M-MoS_2_; 2H-MoS_2_	24 h	Cr(VI) 1000 mg/L	200.3 1T-MoS_2_; 82.8 M-MoS_2_; 70.7 2H-MoS_2_	[152]
CuO	2 h	Cr(VI) 20 mg/L	15.625 Cr(VI)	[157]
Organo-modified MTS	30 min	Cr^3+^ 0.2g	13.81 Cr^3+^	[158]
Fe_3_O_4_/SiO_2_/polythiophene	6.8 min	Hg^2+^ 40 μg/L	59 Hg^2+^ (sorption capacity)	[159]
Fe_3_O_4_/PANI/MnO_2_	30 min	Cd^2+^	99.1% (154)	[162]
MnO_2_/ Fe_3_O_4_/PmPD	24 h	Pb^2+^ 1.2 ppm, Cd^2+^	438.6 Pb^2+^;121.5 Cd^2+^	[164]
PPy–mPD/Fe_3_O_4_	24 h	Cr(VI) 100-600mg/L	99.6% (25 °C; 100mg/L Cr(VI)); 555.6 (maximum adsorption capacity)	[167]
EDTA/ Fe_3_O_4_/SC	2 h	Cd^2+^ 30 mg/L	63.3 Cd^2+^	[169]
nZVN	5 h	Cr(VI)	Cr(VI) (98%), 60.0 (adsorption capacity); 0.6699/min (26 °C); 0.7956/min (30 °C); 1.0251/min (36 °C)	[173]
PPy/Fe_3_O_4_/rGO	12 h	Cr(VI)	293.3 Cr(VI)	[177]
rGO/Fe_3_O_4_ (PANI)	5 h	Hg^2+^ 125.45mg/L	375 Hg^2+^	[180]
MGO/SiO_2_/CN	40 min	Pb^2+^	111.11 Pb^2+^	[182]
MWCNTs	2 h	Hg^2+^ 0.1 mg/L	0.486 Hg^2+^	[185]
MWCNTs (amine and -SH)	5 min	Hg^2+^	84.66 (88.7%)	[186]
e-MWCNTs	30 min	Cd^2+^ 5 mg/L	25.7 Cd^2+^	[190]
PSF-GO	10.5 h Cr^6+^ 12 h Cd^2+^ 13 h Cu^2+^ 15 h Pb^2+^	Pb^2+^, Cu^2+^, Cd^2+^, Cr(VI) 500 mg/L	78.50 Pb^2+^, 68.30 Cu^2+^, 75.60 Cd^2+^, 159.50 Cr(VI)	[193]
s-PBC/GO	180 min	(1000mg/L)	74.2 Co^2+^, 93 Ni^2+^, 229.4 Pb^2+^,	[194]
MOF/iNPs	3 h	As^3+^, As(V), Cd^2+^, Cr^3+^, Cu^2+^, Pb^2+^ and Hg ^2+^	Cu^2+^ 65%; Cr^3+^ 99% ; Cu^2+^ 99% ;Pb^2+^ 99%; Hg^2+^ 99% ; As(V) 86% ;	[198]
CNCs-g-nBA	360 min	Pb^2+^ 125 mg/L	140.95 Pb^2+^	[201]
Nanofiber (PVA/SA ratios (40/60))	100 min	Cd^2+^ 40 ppm	Cd^2+^ 67.05 (max)	[208]
T/G	30 min	As^3+^	As^3+^ 98%(T)	[212]

Abbreviations: Metallic 1T phase of MoS_2_ (1T- MoS_2_), 1T/2H mixed phase MoS_2_ (M- MoS_2_), micelle templated silicas (MTS), polyaniline (PANI), polypyrrole/m-phenyl diamine (PPy–mPD), sawdust composites (SC), nano zerovalent nickel (nZVN), reduced graphene oxide (rGO), polypyrrole (PPy), cyanogen (CN), ethylenediamine-functionalized MWCNTs (e-MWCNT), polysulfone (PSF), sulfonated pentablock copolymer (s-PBC), metal–organic framework (MOF), inorganic nanoparticles (iNPs), cellulose nano crystals (CNCs), butyl acrylate (BA), nano crystals (CNCs)-graft-n butyl acrylate (CNCs-g-nBA), titanate (T), alginate (G).

## 4. Conclusions and Perspectives

Representative progress in the nanotechnology-based analysis and removal of heavy metals has been summarized in this review. For heavy metal analysis, nanomaterials can be incorporated as different roles of (1) adsorbent, (2) filter membrane, (3) reducing agent, (4) peroxide catalyst, and (5) conjugator. The detection can be based on electrochemical, colorimetrical, fluorescent, and biosensing technologies. Various nanomaterials, such as carbon-based, magnetic, semiconducting, and noble metal nanoparticles, have been introduced and discussed. For heavy metal removal, magnetic nanoparticles are ideal adsorbents since they can be easily recovered and collected by an external magnet. On the contrary, using other nanomaterials (that with no magnetism) to separate targets from complex matrices involves cumbersome procedures, such as filtration or centrifuge, which inevitably increase the separation costs.

Nanocomposite materials exhibit outstanding performance in both analysis and removal of heavy metals. As heavy metal nanosensors, such materials have advantages of wide linear range, low detection limit, high sensitivity, and good selectivity. However, since they are in the early stages of development, they currently have defects of (1) fluctuated stability, (2) low practicability in field, (3) high synthetic price, and (4) complex synthetic procedures. As heavy metal nanoremovers, nanocomposites are drawing concerns about the toxicity of some interface materials (including mercury and QDs), especially when they are designed for food, water, and other environmental samples. Current safety standards of nanomaterials are suggested to be reviewed on a relatively frequent basis, in order to keep up with the emerging field. More studies on the biocompatibility of nanomaterials with human health as well as the environment are needed.

In summary, nanotechnology has been evolving into a significant approach for heavy metal analysis and removal from food and water. It is expected that nanosensors and nanoremovers can be evolved for easy and accurate field operation, with balanced properties of high performance, low cost, and great portability.
